# RGB-D Visual SLAM Based on Yolov4-Tiny in Indoor Dynamic Environment

**DOI:** 10.3390/mi13020230

**Published:** 2022-01-30

**Authors:** Zhanyuan Chang, Honglin Wu, Yunlong Sun, Chuanjiang Li

**Affiliations:** 1 College of Information, Mechanical and Electrical Engineering, Shanghai Normal University, Shanghai 200234, China; 1000496110@smail.shnu.edu.cn (H.W.); licj@shnu.edu.cn (C.L.); 2 China North Vehicle Research Institute, Beijing 100072, China; sunyunlong0124@sina.cn

**Keywords:** visual SLAM, LK optical flow, object detection, epipolar geometric constraints, Yolov4-Tiny

## Abstract

For a SLAM system operating in a dynamic indoor environment, its position estimation accuracy and visual odometer stability could be reduced because the system can be easily affected by moving obstacles. In this paper, a visual SLAM algorithm based on the Yolov4-Tiny network is proposed. Meanwhile, a dynamic feature point elimination strategy based on the traditional ORBSLAM is proposed. Besides this, to obtain semantic information, object detection is carried out when the feature points of the image are extracted. In addition, the epipolar geometry algorithm and the LK optical flow method are employed to detect dynamic objects. The dynamic feature points are removed in the tracking thread, and only the static feature points are used to estimate the position of the camera. The proposed method is evaluated on the TUM dataset. The experimental results show that, compared with ORB-SLAM2, our algorithm improves the camera position estimation accuracy by 93.35% in a highly dynamic environment. Additionally, the average time needed by our algorithm to process an image frame in the tracking thread is 21.49 ms, achieving real-time performance.

## 1. Introduction

Traditional visual simultaneous localization and mapping (SLAM) systems can achieve good results in static and rigid scenes with no obvious changes. However, in dynamic scenes, neither the feature-based SLAM algorithm nor the SLAM algorithm based on the direct method can distinguish the feature types of moving object areas. The matching point pairs of dynamic feature points in these scenes will produce data error associations, which will directly reduce the pose estimation accuracy of the visual odometer and lose the pose tracking of the camera. This greatly limits the application of many excellent visual SLAM algorithms. To solve the above problems, the research on visual SLAM in dynamic scenes has attracted much attention, and it has become a research hotspot [1].

At present, there are mainly two methods to detect dynamic obstacles in a scene; one includes traditional geometry-based methods, such as the background subtraction method, the inter-frame difference method, the optical flow method, etc. The geometry method seeks to operate the pixels in the image, which has high accuracy for moving object detection, but it also leads to the problems of high computational consumption and low real-time performance. With the development of computer vision and deep learning, many researchers have begun to apply the semantic information extracted from images to the visual SLAM system, such as by establishing semantic maps, and removing the objects that can move in the environment. Detecting and removing dynamic objects in the process of SLAM through the deep learning method can greatly improve the performance of SLAM systems. There are still two main problems in these methods; one is that powerful semantic segmentation networks such as Mask-RCNN are highly computationally expensive and not applicable to real-time and small-scale robotic applications. The other is that the deep neural network can only get dynamic objects that are known, and they cannot detect objects that without prior knowledge [2,3].

Aiming at the problems of the low accuracy and poor real-time performance of the existing visual SLAM systems in a dynamic environment, this paper combines the lightweight network Yolov4-Tiny with the ORB-SLAM2 algorithm. Meanwhile, the Epipolar geometry constraint and the LK optical flow method are also introduced to filter the possible residual dynamic feature points from the image. Besides this, only static feature points are used for feature matching to solve the pose of the camera so that the influence of dynamic objects on the SLAM system can be eliminated. Experimental verification on the open TUM dataset has shown good results.

## 2. Related Work

### 2.1. Dynamic SLAM Based on Geometric Method

Sun et al. [4] used the method of determining the difference between adjacent frames to detect moving targets, but this method has poor real-time performance. Wang et al. [5] proposed an indoor moving target detection scheme. Firstly, the matched outer points in adjacent frames are filtered through epipolar geometry, and then the clustering information of the depth map provided by the rgb-d camera is fused to identify independent moving targets in the scene. However, the accuracy of the algorithm depends on the pose transformation matrix between adjacent frames. In highly dynamic scenes, the error of the algorithm is large. Lin et al. [6] proposed a method to detect moving objects in a scene using depth information and visual ranging. By fusing the detected outer point information with the depth information of the visual sensor, the position of the moving target in the scene can be easily obtained. However, due to the uncertainty of depth information and the calculation error of the transformation matrix between adjacent frames, the accuracy of target detection and segmentation is low.

The above methods are based on the same principle: the moving object part in the image is regarded as an outlier, which is excluded in the process of estimating attitude, meaning this only depends on the static part of the scene. As a result, the accuracy of current estimation methods depends on the proportion of static feature points in the scene. If there are too many dense dynamic objects in the scene, the reliability of pose estimation will be seriously affected, and the accuracy of map construction will be affected.

### 2.2. SLAM Based on Deep Learning or Semantic Information

In recent years, with the development of deep learning, deep learning technology is being combined with SLAM algorithms to deal with dynamic obstacles in an indoor dynamic environment. Chao Yu et al. [7] proposed DS-SLAM based on the ORB-SLAM2 framework, which uses the SegNet network to obtain semantic information in the scene with independent threads. Then, the inter-frame transformation matrix is estimated through the RANSAC algorithm, and the pole line geometry is adopted to judge feature point states. When the number of dynamic feature points on an object is greater than the threshold, the object is considered dynamic, and all feature points are filtered. This algorithm performs well on the TUM dataset. However, since the basic matrix used in the polar constraint is calculated based on all feature points, the estimated basic matrix will suffer from serious deviations when there are too many abnormal feature points in the image. Similarly, Berta Bescos et al. [8] proposed a DynaSLAM algorithm based on ORB-SLAM2, which filters out dynamic feature points in scenarios by combining geometry and deep learning. The algorithm achieves excellent results on the TUM dataset, but mask-RCNN cannot be used in real time, which affects the application of this algorithm in a real environment. DDL-SLAM [9] detects dynamic objects with semantic masks obtained by DUNet and multi-view geometry, and then reconstructs the background that is obscured by dynamic objects with the strategy of image inpainting. Given that the computation of the masks of dynamic objects is a process taking place at the pixel level, this method also cannot achieve real-time performance. Y. Fan et al. [10] proposed a semantic SLAM system by using BlitzNet to obtain the masks and bounding boxes of dynamic objects in images. The images can be quickly divided into environment regions and dynamic regions, and the depth-stable matching points in the environment are used to construct epipolar constraints to locate the static matching points in the dynamic regions. However, the method still has two problems; one is the real-time problem, and the other is that the method cannot solve unknown objects. Han and Xi [11] proposed a PSPnet-SLAM (Pyramid Scene Parsing Network–SLAM) to improve ORB-SLAM2, in which the PSPNet and optical flow are used to detect dynamic characteristics. The features extracted from labeled dynamic objects and the features with large optical flow values are filtered out, and the rest are used for tracking. This method achieves high positioning accuracy. Zhang et al. [12] used Yolo running in an independent thread to acquire semantic information, assuming that features extracted from moving objects would be unstable and need to be filtered out. Li et al. [13] also used Yolo to detect dynamic features, and they proposed a novel sliding window compensation algorithm to reduce the detection errors of Yolo, thus providing a new means of detecting dynamic objects.

## 3. System Overview

### 3.1. Algorithm Framework

In a dynamic environment, the ORB-SLAM2 algorithm is affected by moving objects, which results in reduced positioning accuracy and poor robustness. To address this issue, this paper introduces an object detection thread to detect moving objects based on the original ORB-SLAM2 algorithm. Meanwhile, the lightweight object detection network Yolov4-Tiny is used to detect objects in the input image while extracting feature points at the front end. After the semantic information in the image is obtained, the dynamic objects in the image are determined. According to the object detection results, a module that can remove the dynamic feature points is added to the tracking thread.

**Figure 1:**
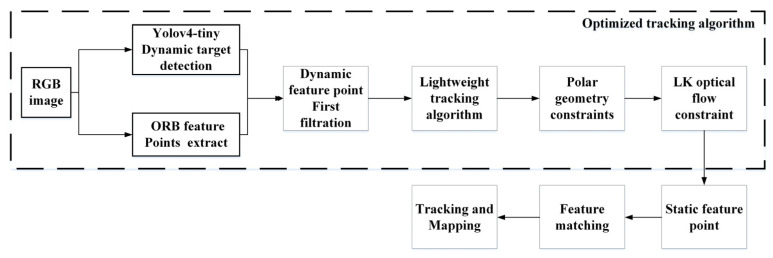
The flow chart of the improved tracking algorithm in this paper.

Because some potential dynamic objects in the environment and some blurred moving objects in the image may not be detected by the object detection network, the quasi-static feature points derived after the first filtering will be used to match the feature points. Then, according to the feature point matching results, the essential matrix between two images can be obtained by the RANSAC algorithm. Next, the epipolar geometric constraints and LK optical flow method can be adopted in series to detect and remove the potential dynamic feature points. Finally, only the remaining static feature points are used to estimate the pose between adjacent frames. The flow chart of the improved algorithm discussed in this paper is shown in Figure 1.

### 3.2. Yolov4-Tiny

**Figure 2:**
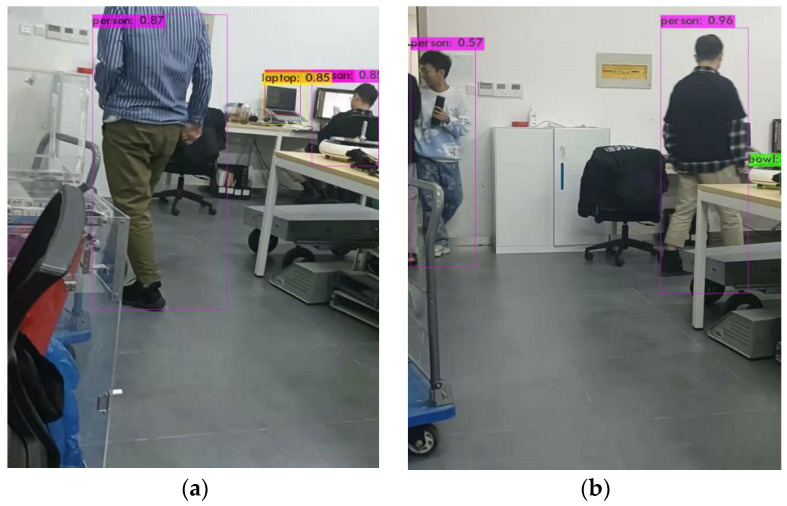
Object detection results of Yolov4-Tiny. (**a**) Results 1; (**b**) Results 2.

**Figure 3:**
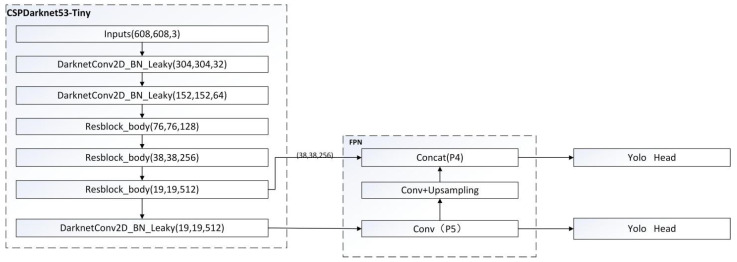
Yolov4-Tiny network structure.

The YOLOv4-tiny structure is a simplified version of YOLOv4, which is a lightweight model. There are only 6 million parameters, which is equivalent to one-tenth of the original, which greatly improves the detection speed. The overall network structure has a total of 38 layers, using three residual units. The activation function uses LeakyReLU, the classification and regression of the target are modified to use two feature layers, and the feature pyramid (FPN) network is used when merging the effective feature layers. It also uses the CSPnet structure, and performs channel segmentation on the feature extraction network. The feature layer channel output after 3 × 3 convolutions is divided into two parts, and the second part is used. On the COCO dataset, 40.2% AP50 and 371FPS were obtained, indicating a significant performance advantage over other versions of lightweight models. Yolov4-Tiny object detection network is used for object detection experiment, and the results are shown in Figure 2. The structural diagram of this model is shown in Figure 3 below [14,15].

### 3.3. Backbone Network Structure of Yolov4-Tiny

**Figure 4:**
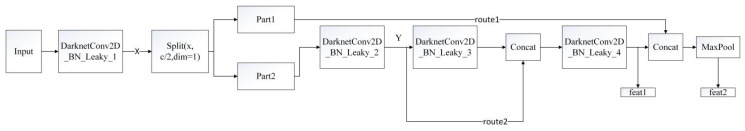
The structure of Resblock_body.

As shown in Figure 3, the backbone network of Yolov4-Tiny, i.e., CSPDarknet53-Tiny, is composed of the Resblock_body module and the DarknetConv2d_BN_Leaky module. DarknetConv2d_BN_Leaky mainly contains of a two-dimensional convolution module, and the standardization and activation function Leaky ReLU. The CSPNet structure is introduced into the Resblock_body module as shown in Figure 4. The trunk part of the module is still the conventional stacking of residual modules, but a long-span residual edge is introduced in the branch part. The residual edge is first processed by a small amount of convolution, and then directly connected to the module. Next, it is concatenated with the output of the trunk part in the channel dimension. Finally, the output of the module is processed by the 2 × 2 maximum pooling layer.

The CSPNet structure can reduce the number of network parameters by 10~30%, and thus ensure that the accuracy of the model is unchanged or slightly improved. In Figure 4, feat1 and feat2 are the outputs of the initial feature layer in the Resblock_body module. For the first two Resblock_body modules in the backbone feature network, the initial feature layer, feat1, will be discarded directly, while feat2 will be used as the input feature of the latter Resblock_body. For the third Resblock_body, it outputs features feat1 and feat2. Feat1 is used as the first input of the feature enhancement network, and feat2 will be passed through the DarknetConv2d_ BN_Leaky module and processed as the second input for the feature enhancement network.

### 3.4. Dynamic Feature Point Elimination Strategy

#### 3.4.1. Dynamic Feature Point Elimination Based on Object Detection

In the dynamic object prediction box generated based on object detection, the dynamic feature points are determined by prior knowledge, and the specific elimination process is described as follows [9,10,11]:

Denote all feature points of the image extracted by the visual odometer as $P_{k}$ when the *k*-th frame image is input. $P_{k}$ can be expressed as $P_{k}=\{p_{1},p_{2},p_{3},\ldots p_{n}\}$ After the image passes through the object detection network, all dynamic feature points can be determined with prior knowledge as $D_{k}$. According to the semantic information prediction box, $D_{k}$ can be expressed as $D_{k}=\{d_{1},d_{2},d_{3},\ldots d_{n}\}$. The above description indicates that if $p_{i}\in D_{k}\left(i=1,2,3,\ldots n\right)$, then the feature point $p_{i}$ is considered as a dynamic feature point, and it is then removed from $P_{k}$ in the tracking thread. The remaining feature points are quasi-static feature points, and the set of these points is denoted as $S_{k}$. We have $S_{k}\cup D_{k}=P_{k}$.

#### 3.4.2. Epipolar Geometry Constraints

**Figure 5:**
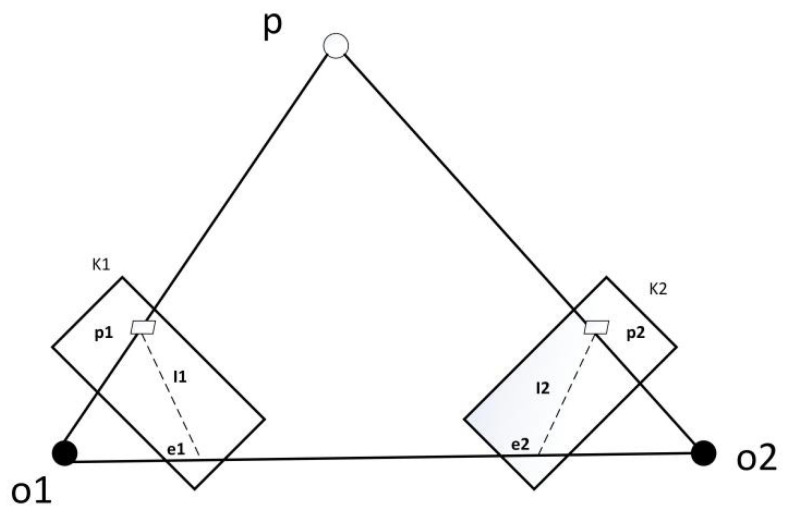
Epipolar geometry constraints.

After the first step of object detection, the set of quasi-static feature points $S_{k}$ can be obtained, and then the current frame and the reference frame are used for feature matching to obtain the set of matching point pairs Q. The basic matrix F can be calculated by the RANSAC algorithm using set Q. The specific calculation process is as follows:

As shown in Figure 5, firstly, denote the pair of matching points in the current frame and the reference frame as $p_{1}$ and $p_{2}$, respectively. $P_{1}$ and $P_{2}$ are the corresponding homogeneous coordinate forms:

(1) $$\begin{array}{l} p_{1}=\left[u_{1},v_{1}\right],p_{2}=\left[u_{2},v_{2}\right]\\ P_{1}=\left[u_{1},v_{1},1\right],P_{2}=\left[u_{2},v_{2},1\right] \end{array}$$

u and v respectively represent the coordinate values of the feature points in the image pixel coordinate system. Then, denote the pole line of each frame as $I_{i}$. The calculation formula of $I_{i}$ is shown as follows.

(2) $$I_{i}=\left[\begin{array}{l} X\\ Y\\ Z \end{array}\right]=FP_{i}=F\left[\begin{array}{l} u_{i}\\ v_{i}\\ 1 \end{array}\right]\left(i=1,2\right)$$

where X, Y and Z are line vectors, and F is the essential matrix. Then, the distance D between the matching point pair and the corresponding polar line can be calculated by the following formula:

(3) $$D=\frac{\left|P_{2}{}^{T}FP_{1}\right|}{\sqrt{{\left\|X\right\|}^{2}+{\left\|Y\right\|}^{2}}}$$

According to Formula (3), the distance *d* from each quasi-static feature point to the polar line can be obtained. Then, D is compared with the preset threshold ε. If D>ε, this point is considered to be an external point and should be filtered out in the tracking stage [16,17,18,19,20,21].

When the motion direction of the moving object is parallel to the camera, the polar geometric constraints can also be satisfied. In this case, the antipolar geometric constraints are not applicable. Here, the LK optical flow method is adopted to further detect the dynamic objects in the environment and filter the dynamic feature points for the third time [22,23,24].

#### 3.4.3. LK Optical Flow Constraint

The LK optical flow method is based on three assumptions: (1) the pixel brightness in the image does not change between successive frames; (2) the time interval between frames is relatively short; (3) adjacent pixels have similar motions [25,26,27]. According to assumption 1, the gray level is constant, and it can be obtained via:

(4) I(x,y,t)=I(x+dx,y+dy,t+dt)

where t and t+dt are the corresponding times of adjacent image frames. I(x,y,t) and I(x+dx,y+dy,t+dt) are the positions of the pixel points in the image.

According to assumption 2, due to the small time interval between adjacent image frames, Taylor series expansion on the right side of (4) can be enacted to obtain:

(5) $$I\left(x+dx,y+dy,t+dt\right)\approx I\left(x,y,t\right)+\frac{\partial I}{\partial x}dx+\frac{\partial I}{\partial y}dy+\frac{\partial I}{\partial t}dt$$

By combining (4) and (5), we have

(6) $$\frac{\partial I}{\partial x}dx+\frac{\partial I}{\partial y}dy+\frac{\partial I}{\partial t}dt=0$$

Then, dividing both sides of (6) by dt we have

(7) $$\frac{\partial I}{\partial x}\frac{dx}{dt}+\frac{\partial I}{\partial y}\frac{dy}{dt}=-\frac{\partial I}{\partial t}$$

$\frac{dx}{dt}$ and $\frac{dy}{dt}$ are respectively the velocity of the feature point on the *x*-axis and on the *y*-axis, and they are denoted as u and v. Denote $\frac{\partial I}{\partial x}$ as $I_{x}$, $\frac{\partial I}{\partial y}$ as $I_{y}$, and the change in the gray level of the feature point with time as $I_{t}$, and the following result can be obtained by writing (7) in matrix form:

(8) $$\left[I_{x}I_{y}\right]\left[\begin{array}{l} u\\ v \end{array}\right]=-I_{t}$$

However, additional constraints need to be introduced in the LK optical flow to calculate the velocity u and v of the feature points. According to assumption 3, i.e., adjacent pixels have similar motions, a 3 × 3 window centered on the feature point is selected, and nine pixels within the window have the same motion. The equation in (9) can be performed for nine pixels simultaneously:

(9) $${\left[I_{x}I_{y}\right]}_{k}\left[\begin{array}{l} u\\ v \end{array}\right]=-I_{tk}\left(k=1,2,3,...9\right)$$

Denoting $\left[\begin{array}{l} I_{x1}I_{y1}\\ I_{x2}I_{y2}\\ \vdots \vdots \\ I_{x9}I_{y9} \end{array}\right]$ as A, $\left[\begin{array}{l} u\\ v \end{array}\right]$ as V, and $\left[\begin{array}{l} -I_{t1}\\ -I_{t2}\\ \vdots \\ -I_{t9} \end{array}\right]$ as b, the least square method is adopted to solve (9), and the results are as follows:

(10) $$A^{T}AV=A^{T}b$$

(11) $$V={\left(A^{T}A\right)}^{-1}A^{T}b$$

**Figure 6:**
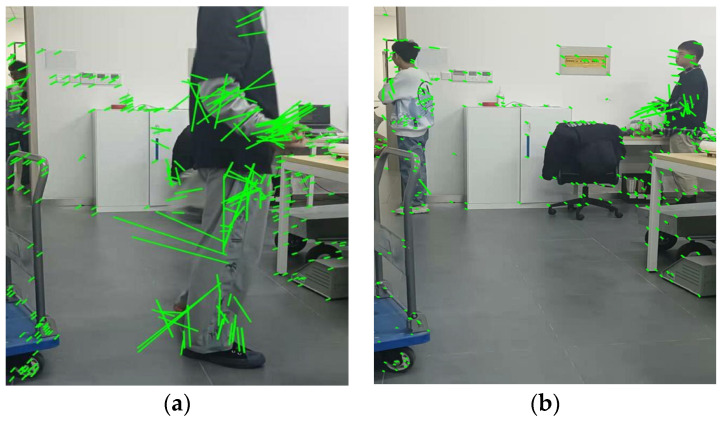
The results of using the optical flow method to detect dynamic feature points. (**a**) Result 1; (**b**) Result 2.

The size of the optical flow is calculated for the quasi-static feature points after object detection under polar geometry constraints. Then, by solving its mean value and standard deviation, (12) and (13) can be used to determine whether the feature point is a dynamic feature point.

(12) $$\left|L_{i}-L_{avg}\right|>2L_{std}$$

(13) $$\left|L_{i}-L_{avg}\right|>L_{thr1}\left(L_{std}<L_{thr2}\right)$$

where $L_{i}$ is the optical flow size of the *i*-th feature point; $L_{avg}$ and $L_{std}$ are, respectively, the mean and standard deviation of the optical flow size of all feature points; $L_{thr1}$ and $L_{thr2}$ are preset thresholds. If the optical flow size $L_{i}$ of the *i*-th feature point meets the above relationship, the special diagnosis point *i* is determined to be a dynamic feature point. As we can see in Figure 6, we can find the difference in optical flow size between the dynamic feature points and static feature points; the green line is the high optical flow, and the green point is the normal optical flow.

**Figure 7:**
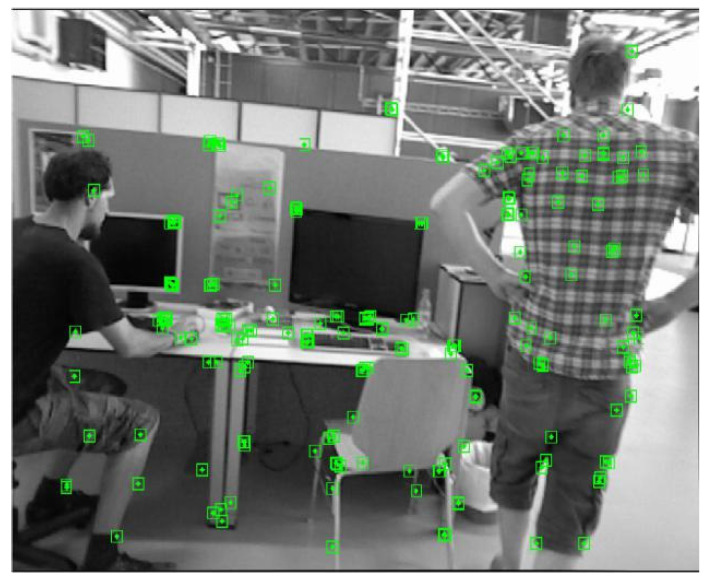
Dynamic feature points were not eliminated.

**Figure 8:**
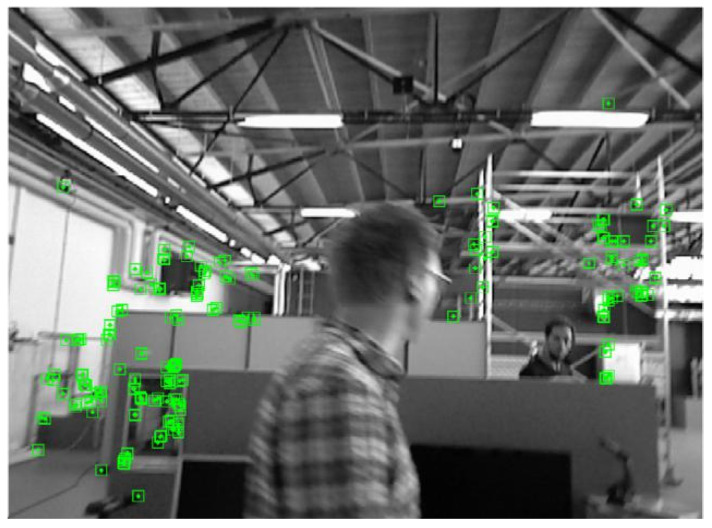
Results of dynamic feature points’ removal.

Because Yolov4-Tiny is a lightweight network, part of the detection accuracy is sacrificed to improve the running speed of the algorithm. To make up for the loss of detection accuracy, this paper introduces pole-constrained geometry and the optical flow method, which are introduced to further detect the dynamic objects in the environment and filter out dynamic feature points thoroughly. Figure 7 and Figure 8 below illustrates the filtering of dynamic objects from the freiburg3_Walking_xyz highly dynamic scene sequence in the TUM dataset. One of the men in the picture is walking randomly [28,29,30]. Figure 7 is the result of traditional orb-slam2 without dynamic feature point filtering, and Figure 8 is the result of dynamic feature point filtering.

## 4. Results

### 4.1. Experimental Data Sets

In the experiment, the mainstream public dataset TUM RGB-D was used to evaluate the performance of the SLAM algorithm proposed in this paper. This dataset is a standard RGB-D dataset provided by the Computer Vision Class group of Technical University of Munich, Germany, and it has been used by many scholars in the SLAM research field to evaluate the performance of SLAM algorithms [30,31,32,33,34]. The data in the dataset are mainly divided into the low dynamic scene dataset fr3_sitting_xx and the high dynamic scene dataset fr3_walking_xx. The real trajectory of the dataset was captured by a high-precision motion capture system. The capture system was composed of eight high-speed cameras and an inertial measurement system, which captured the real position and pose data from the camera in real time.

The experimental equipment used in this experiment was a Lenovo Savior R7000 laptop; its CPU model is R7-4800H, its main frequency is 2.9 GHz, its graphics card is an NVDIA Geforce RTX2060, and its system environment is Ubuntu18.04.

### 4.2. Analysis of the Experimental Results

In this paper, six datasets from the TUM dataset are used for experimental verification, including fr3_walking_xyz, fr3_walking_half, fr3_walking_static, fr3_walking_rpy, fr3_sitting_static and fr3_sitting_half. The datasets of walking_xx belong to a highly dynamic environment, and the datasets of sitting_xx belong to a low dynamic environment. The Evo tool is used to compare the camera pose estimated by the proposed algorithm with the real camera pose data provided in the dataset. The evaluation indicator is Absolute Pose Error (APE). The APE refers to the direct difference between the pose of the camera estimated by the algorithm and the real pose data. It directly reflects the accuracy of pose estimation and the consistency of the global trajectory of the camera. Meanwhile, Root Mean Square Error (RMSE) and Standard Deviation (STD) are used for evaluation. RMSE reflects the difference between the real value and the observed value. STD is used to evaluate the deviation between the camera’s estimated trajectory and the real trajectory, which can reflect the robustness and stability of the system. The mean and median can reflect the accuracy of pose estimation. For performance comparison, our algorithm is compared with the use of ORB-SLAM2 for the same dataset, and the experimental results are as follows.

In the table below, each group of experiments is carried out four times, and the results are averaged. The relative improvement rate η is calculated by the following formula:

(14) $$\eta =\frac{orbslam2-ours}{orbslam2}\times 100\%$$

**Figure 9:**
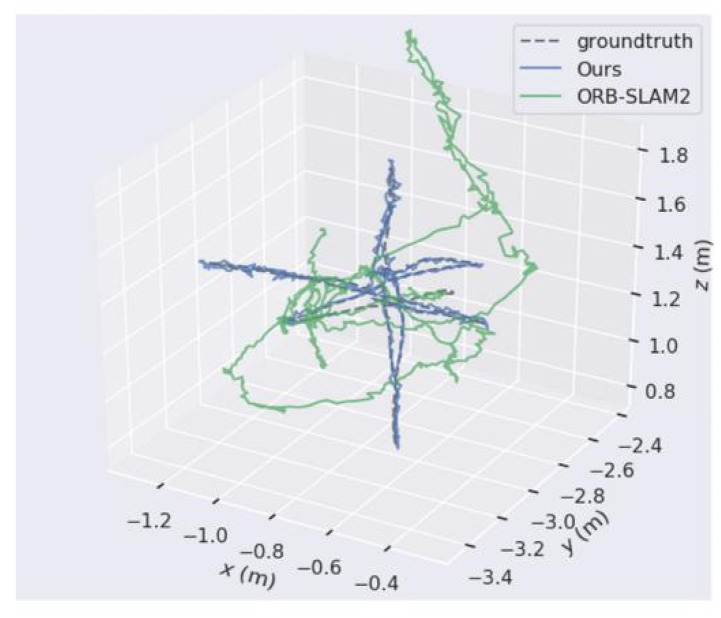
The comparison of our algorithm’s trajectory with ORB-SLAM2’s when applied on walking_xyz from the TUM dataset.

**Figure 10:**
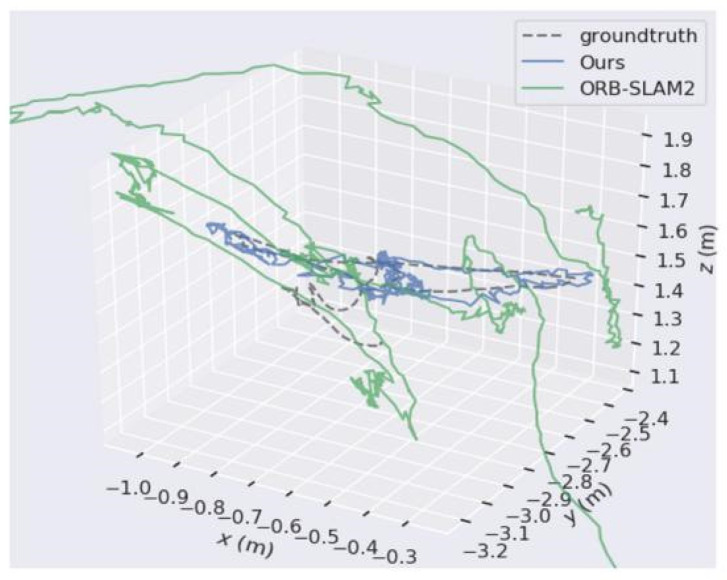
The comparison of our algorithm’s trajectory with ORB-SLAM2’s when applied on walking_rpy from the TUM dataset.

Figure 9 and Figure 10, respectively, show the trajectory distribution obtained by the algorithm proposed in this paper and by ORB-SLAM2 applied on walking_xyz and Walking_rpy from the TUM dataset. Compared with ORB-SLAM2, the trajectory of the camera estimated by our algorithm is closer to the real trajectory of the camera.

**Figure 11:**
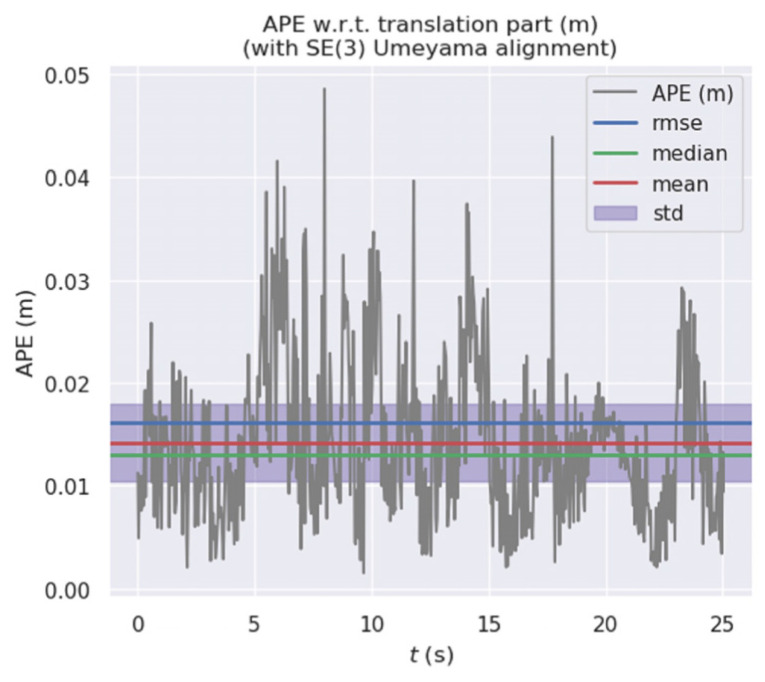
Error distribution graph of our algorithm for the walking_xyz dataset.

**Figure 12:**
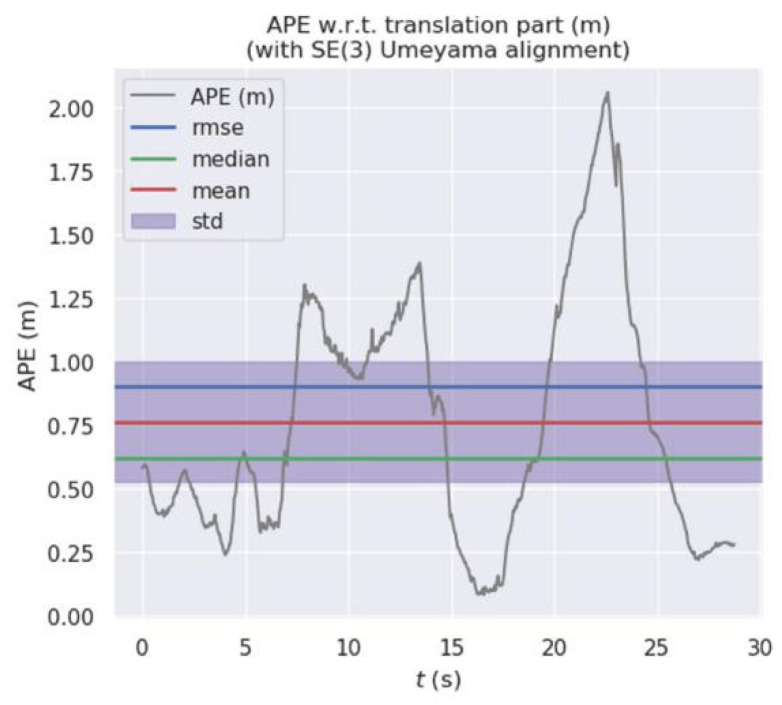
Error distribution graph of ORB-SLAM2 for the walking_xyz dataset.

**Figure 13:**
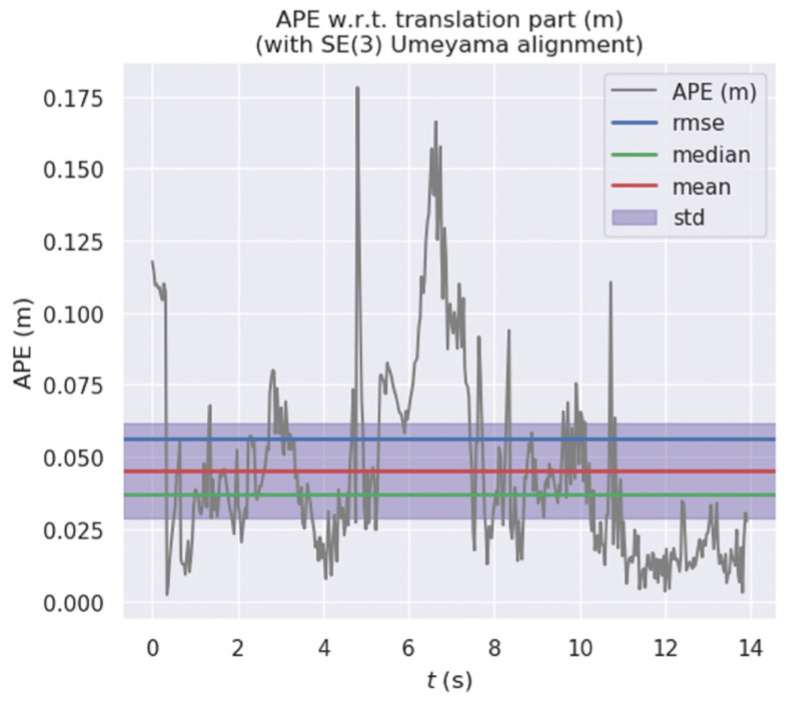
Error distribution graph of our algorithm for the walking_rpy dataset.

**Figure 14:**
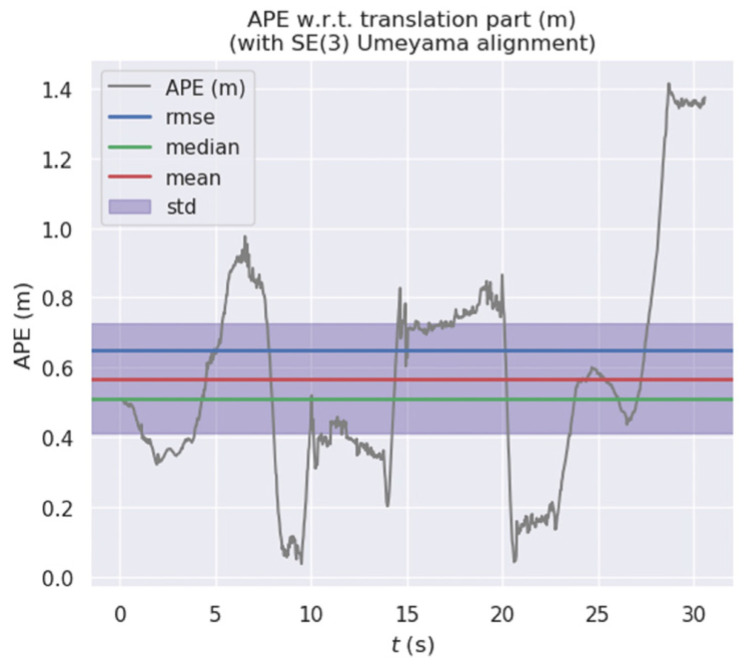
Error distribution graph of ORB-SLAM2 for the walking_rpy dataset.

Figure 11 and Figure 12 show the error distribution of the results obtained by our algorithm and ORB-SLAM2 when applied on walking_xyz. Figure 13 and Figure 14 show the error distribution of the results obtained by our algorithm and ORB-SLAM2 for walking_rpy. Compared with the ORB-SLAM2 algorithm, our algorithm reduces the error values by an order of magnitude.

The results in Table 1 show that, compared with the classic ORB-SLAM2 algorithm, the algorithm proposed in this paper achieves an average RMSE improvement of 93.35% in a highly dynamic environment. However, in a low dynamic environment, the improvement of the RMSE by our algorithm is not too high compared with that of ORB-SLAM2, which indicates that the traditional ORB-SLAM2 algorithm can achieve a better effect without the interference of dynamic objects. Therefore, the algorithm proposed in this paper can overcome the low accuracy of pose estimation caused by the interference of moving objects in a dynamic environment.

To further verify the performance of the proposed algorithm, this paper also compares it with the ORB-SLAM3algorithm and other algorithms based on deep learning [35]. By analyzing the data in Table 2, it can be determined that among the algorithms listed in this paper, Dyna-SLAM, Ds-SLAM and the algorithm proposed in this paper can achieve the highest positioning accuracy. Among them, Dyna-SLAM and Ds-SLAM use the Mask-RCNN and Segnet semantic segmentation networks to detect dynamic objects in the environment, respectively. Since semantic segmentation is performed pixel by pixel, the detection accuracy will be greater than that of the target detection model used in this paper (Yolo), but the lightweight object detection network Yolov4-Tiny used in this paper is better than the above two algorithms in terms of algorithm execution speed. Table 2 below lists the time required for the three algorithms to process each frame of picture. So, our algorithm achieves a good balance between accuracy and real-time performance, and it can effectively deal with the effects of moving objects on the stability of SLAM systems in a dynamic environment. The comparison results of absolute trajectory error of different algorithms are shown in Table 3.

### 4.3. Discussion and Outlook

By observing the experimental data in Table 1, the improvement effect of the walking_half dataset is not as obvious as that of other datasets, and tracking failure occurs in the experiment on the dataset walking_static. In view of these problems, by analyzing the data in the dataset, we can see that in the dataset walking_half, many images are blurred due to the movement of the camera, and the characters in the images cannot be recognized by the network, which eventually leads to a decrease in the estimation accuracy, and in the Walking_static dataset, characters occupy most of the images. When the dynamic objects are removed, the remaining static feature points are reduced, and eventually, the tracking fails.

In future work, we will attempt to optimize the target detection model to improve the detection accuracy while keeping the speed constant, so that the model can more accurately identify dynamic objects in the environment and eliminate the impact of dynamic objects on the SLAM system. In addition, we will use the extracted semantic information to build dense maps to help the system in navigation and obstacle avoidance tasks at a later stage.

## 5. Conclusions

In an indoor dynamic environment, SLAM systems are prone to be affected by moving objects, which may reduce the pose estimation accuracy and cause tracking failures. In this paper, a SLAM algorithm based on the classic ORB-SLAM2 framework in an indoor dynamic environment is proposed. The object detection network Yolov4-Tiny is used to detect the dynamic semantic objects in the environment. Then, the dynamic feature points are filtered out before they are tracked, and only the static feature points are used for pose estimation. Experimental verification on the TUM dataset shows that compared with the classic ORB-SLAM2 algorithm, our algorithm reduces the absolute trajectory error by 93.35% in an indoor highly dynamic environment with pedestrians walking back and forth. Additionally, our algorithm only needs 21.49 ms on average to process an image frame in the tracking thread, which can meet the requirements of real-time processing. Compared with other algorithms of the same type, the algorithm proposed in this paper has certain advantages in terms of precision and real-time performance.

## Figures and Tables

**Table 1:** Comparison of absolute pose error between ORB-SLAM2 and our algorithm.

Sequences	ORB-SLAM2				Ours				Improvements			
	Mean	Median	RMSE	STD	Mean	Median	RMSE	STD	Mean	Median	RMSE	STD
Walking_static	0.0966	0.0877	0.1136	0.0598	0.0061	0.0050	0.0074	0.0042	93.68%	94.29%	93.48%	92.97%
Walking_xyz	0.5478	0.6111	0.6015	0.2485	0.0142	0.0130	0.0160	0.0074	97.40%	97.87%	97.33%	97.02%
Walking_rpy	0.6026	0.5556	0.7010	0.3581	0.0453	0.0368	0.0561	0.0331	92.48%	93.37%	91.99%	90.75%
Walking_half	0.4272	0.3964	0.4863	0.2290	0.0413	0.0369	0.0458	0.0197	90.33%	90.69%	90.58%	91.39%
Sitting_half	0.0167	0.0147	0.0190	0.0092	0.0251	0.0263	0.0279	0.0123	33.46%	44.10%	31.89%	25.20%
Sitting_static	0.0074	0.0064	0.0085	0.0041	0.0065	0.0058	0.0074	0.0035	12.16%	9.37%	12.94%	14.63%

**Table 2:** Tracking time comparison (ms).

Algorithm	Time
Dyna-SLAM	900
Ds-SLAM	200
Ours	21.49

**Table 3:** The absolute trajectory error of different algorithms.

Sequences	ORB-SLAM3	Dyna-SLAM	Ds-SLAM	DVO-SLAM	OFD-SLAM	Ours
Walking_static	0.0203	0.0090	0.0081	--	--	0.0074
Walking_xyz	0.2341	0.0150	0.0247	0.5966	0.1899	0.0160
Walking_rpy	0.1552	0.0400	0.4442	0.7304	0.1533	0.0561
Walking_half	0.4372	0.0250	0.0303	0.5287	0.1612	0.0458
Sitting_static	0.0089	0.0065	0.0064	0.0505	0.0134	0.0074
Sitting_half	0.0335	0.0191	0.0148	--	0.0257	0.0279

## Abbreviations

The following abbreviations are used in this manuscript:

SLAM	Simultaneous Localization and Mapping
LK	Lucas–Kanade
